# Leaf morphometric analysis and potential distribution modelling contribute to taxonomic differentiation in the *Quercus microphylla* complex

**DOI:** 10.1007/s10265-023-01495-z

**Published:** 2023-09-23

**Authors:** Oscar Ángel De Luna-Bonilla, Susana Valencia-Á, Guillermo Ibarra-Manríquez, Saddan Morales-Saldaña, Efraín Tovar-Sánchez, Antonio González-Rodríguez

**Affiliations:** 1 Instituto de Investigaciones en Ecosistemas y Sustentabilidad, Universidad Nacional Autónoma de México, 58190 Morelia, México; 2 Posgrado en Ciencias Biológicas, Unidad de Posgrado, Edificio A, 1° Piso, Circuito de Posgrados, Ciudad Universitaria, Coyoacán, 04510 Ciudad de Mexico, México; 3 Herbario de la Facultad de Ciencias, Departamento de Biología Comparada, Universidad Nacional Autónoma de México, Circuito Exterior, s.n, Ciudad Universitaria, Coyoacán, 04510 México City, México; 4 Centro de Investigación en Biodiversidad y Conservación, Universidad Autónoma del Estado de Morelos, Av. Universidad 1001, Col. Chamilpa, CP, 62209 Cuernavaca, Morelos Mexico

**Keywords:** Morphometrics, Niche analysis, Population approach, Scrub oaks, Taxonomy

## Abstract

Mexico is a major center of evolutionary radiation for the genus *Quercus*, with oak species occurring across different habitat types and showing a wide variation in morphology and growth form. Despite representing about 20% of Mexican species, scrub oaks have received little attention and even basic aspects of their taxonomy and geographic distribution remain unresolved. In this study, we analyzed the morphological and climatic niche differentiation of scrub oak populations forming a complex constituted by six named species, *Quercus cordifolia*, *Quercus frutex*, *Quercus intricata*, *Quercus microphylla*, *Quercus repanda*, *Quercus striatula* and a distinct morphotype of *Q. striatula* identified during field and herbarium work (hereafter named *Q. striatula* II). Samples were obtained from 35 sites covering the geographic distribution of the complex in northern and central Mexico. Morphological differentiation was analyzed through geometric morphometrics of leaf shape and quantification of trichome traits. Our results indicated the presence of two main morphological groups with geographic concordance. The first was formed by *Q. frutex*, *Q. microphylla*, *Q. repanda* and *Q. striatula*, distributed in the Trans-Mexican Volcanic Belt, the Sierra Madre Occidental and a little portion of the south of the Mexican Altiplano (MA). The second group consists of *Q. cordifola*, *Q. intricata* and *Q. striatula* II, found in the Sierra Madre Oriental and the MA. Therefore, our evidence supports the distinctness of the *Q. striatula* II morphotype, indicating the need for a taxonomic revision. Within the two groups, morphological differentiation among taxa varied from very clear to low or inexistent (i.e. *Q. microphylla-Q. striatula* and *Q. cordifolia-Q. striatula* II) but niche comparisons revealed significant niche differentiation in all pairwise comparisons, highlighting the relevance of integrative approaches for the taxonomic resolution of complicated groups such as the one studied here.

**Supplementary Information:**

The online version contains supplementary material available at 10.1007/s10265-023-01495-z.

## Introduction

The genus *Quercus* (Fagaceae) is recognized for its economic and ecological importance and recently has become a model clade in different fields of biology (Cavender-Bares 2019). For these reasons, defining and properly differentiating species in this group is a fundamental task (Valencia-A 2020; Wu et al. 2023). On the basis of phylogenomic analyses (Hipp et al. 2018, 2020), *Quercus* has recently been divided (Denk et al. 2017) into two subgenera with eight sections (subgenus *Quercus*, including sections *Lobatae*, *Ponticae*, *Protobalanus*, *Quercus* and *Virentes*, and subgenus *Cerris*, including sections *Cerris*, *Cyclobalanopsis* and *Ilex*). Mexico is recognized as a major center of diversification of the oaks, with more than 160 species, included in sections *Lobatae, Quercus, Protobalanus* and *Virentes,* and at least 109 endemics (Galicia et al. 2015; Hipp et al. 2018; McCauley et al. 2019; Nixon 1993; Valencia-A 2004).

Despite these recent advances in the clarification of the taxonomy and phylogeny of oaks, considerable confusion remains within groups of closely related species, especially in Mexico (Hipp et al. 2020). Some of these difficulties in the delimitation and identification of oak species arise from phenotypic similarity due to recent divergence, the presence of few taxonomically informative characters in floral traits and the great variation in vegetative characters at different levels (from intraindividual to between populations) (González-Rodríguez et al. 2004; Hardin 1975; Morales-Saldaña et al. 2022; Valencia-A 2004, 2020). Leaves usually display considerable intraspecific variation, since their morphology depends on an intricate relationship between genetic and environmental factors (Chitwood and Sinha 2016; De Heredia et al. 2018). Regarding genetic factors, the frequent gene flow among oak species impacts the expression of morphological characters (Cannon and Petit 2020; Dumolin-Lapégue et al. 1999; Leroy et al. 2017; Whittemore and Schaal 1991). In turn, variation in factors such as light exposure, water availability or temperature, and even pruning, may result in leaf differences between individuals of the same population or between branches of the same individual (Aykut et al. 2017; Borazan and Babaç 2003; Jensen 1990). Therefore, the high degree of leaf polymorphism in oak trees results from the interrelation between introgressive hybridization, phenotypic plasticity, and adaptation to environmental gradients (Morales-Saldaña et al. 2022). In addition to the large intraspecific variation, many of the vegetative characters in *Quercus* also show low interspecific differentiation or tend to evolve in a convergent or parallel manner (Jones 1986; Tucker 1974).

In Mexico, some oak species complexes have been analyzed to demarcate taxa, either from a strictly morphological perspective, as for the *Acutifoliae* group (Romero Rangel 2006) or, more recently, using integrative approaches, as for the *Quercus laeta* complex (Morales-Saldaña et al. 2022) and the *Racemiflorae* group (McCauley et al. 2019). In these recent studies, quantitative methods for analyzing vegetative characters have proven useful for the identification of differentiated groups. Such methods include geometric morphometrics (Albarrán-Lara et al. 2010; Morales-Saldaña et al. 2022; Rodríguez-Gómez et al. 2018; Viscosi 2015; Viscosi et al. 2009b), as well as the statistical characterization of micromorphological structures that are considered of high taxonomic value, such as trichomes (Fortini et al. 2015; López-Caamal et al. 2017).

In comparison to their arboreal congeners, less attention has been given so far to most of the approximately 70 scrub oak species that exist worldwide. At least 31 species with this growth form are distributed in Mexico (21 in section *Quercus*, seven in *Lobatae* and three in *Protobalanus*); 20 of them are endemic and 11 are shared with the USA. In California, studies of scrub oak species have revealed that they frequently form hybrid swarms with adaptations to local environmental conditions, which represents an interesting scenario for the origin of new species (Ortego et al. 2015; Sork et al. 2016). In the case of Mexico, Sabás-Rosales et al. (2017) provided a qualitative analysis of the leaf morphology and trichome types of six scrub oak species. These six species are part of the *Q. microphylla* Née complex, in which all taxa are strictly shrubby in habit, show elliptic, ovate, oblong or lanceolate leaves, mostly with stellate trichomes on the abaxial side or with some stipitate or fasciculate trichomes. Intergradation in leaf morphology is evident among these species (Sabás-Rosales et al. 2017) and, in addition, their area of distribution is not clearly known, mainly because the original descriptions are not sufficiently precise in terms of diagnostic characters, which possibly has caused numerous misidentifications and, consequently, the over- or underestimation of the real distribution of the taxa.

The complex analyzed in this study is named after *Q. microphylla*, a scrub oak species with broad distribution in Mexico (González-Elizondo et al. 1993; Romero Rangel et al. 2002; Sabás-Rosales et al. 2017; Valencia-A 2004; Valencia-A et al. 2017; Vázquez-Villagrán 2000) and problematic taxonomically. The synonymies reported for *Q. microphylla* involve putative varieties of *Q. frutex* Trel. (Valencia-A 2004) and *Q. striatula* Trel. (Bartholomew and Almeda 2023; González Villarreal 1986; McVaugh 1974), problems that have produced a “domino” effect since the inconspicuous differences and apparent sympatric distribution between *Q. microphylla* and *Q. striatula* involve *Q. cordifolia* Trel. too (Muller 1944; Villareal et al. 2008). In the same way, *Q. frutex* may show confusion with *Q. repanda* Bonpl. in distribution and appearance (Romero Rangel et al. 2002, 2014). *Quercus intricata* Trel. adds to the problem by sharing characters such as leaf texture and indumentum appearance with *Q. frutex* and *Q. repanda*, in addition to presenting a sympatric distribution with *Q. cordifolia*. Finally, the complex also includes *Quercus deliquescens* and *Quercus mohoriana*. However, these are geographically restricted species in Mexico, with a distinguishable phenotype from the rest of the complex (Muller 1944; Sabás-Rosales et al. 2017).

In this context, the main objective of this study was to contribute to the recognition of six scrub oak species belonging to the *Q. microphylla* complex (*Q. cordifolia, Q. frutex, Q. intricata, Q. microphylla, Q. repanda* and *Q. striatula*) by: (i) describing the leaf morphology of each species, from a geometric morphometrics approach, (ii) characterizing the types of trichomes present in the species, as well as evaluating if there are differences in the number of trichome arms among the species, and (iii) defining the potential areas of distribution and assessing whether there are niche differences among the species of the *Q. microphylla* complex.

## Materials and methods

### Study species

The six species in this study belong to the section *Quercus* (withe oaks). The six species are shrubby in habit, with rhizomatous growth and with small (0.5 to 2.6 cm) and tomentose to glabrescent leaves. Except for *Q. intricata*, which is also distributed in the southern USA, the rest of the species are endemic to Mexico.

**Fig. 1:**
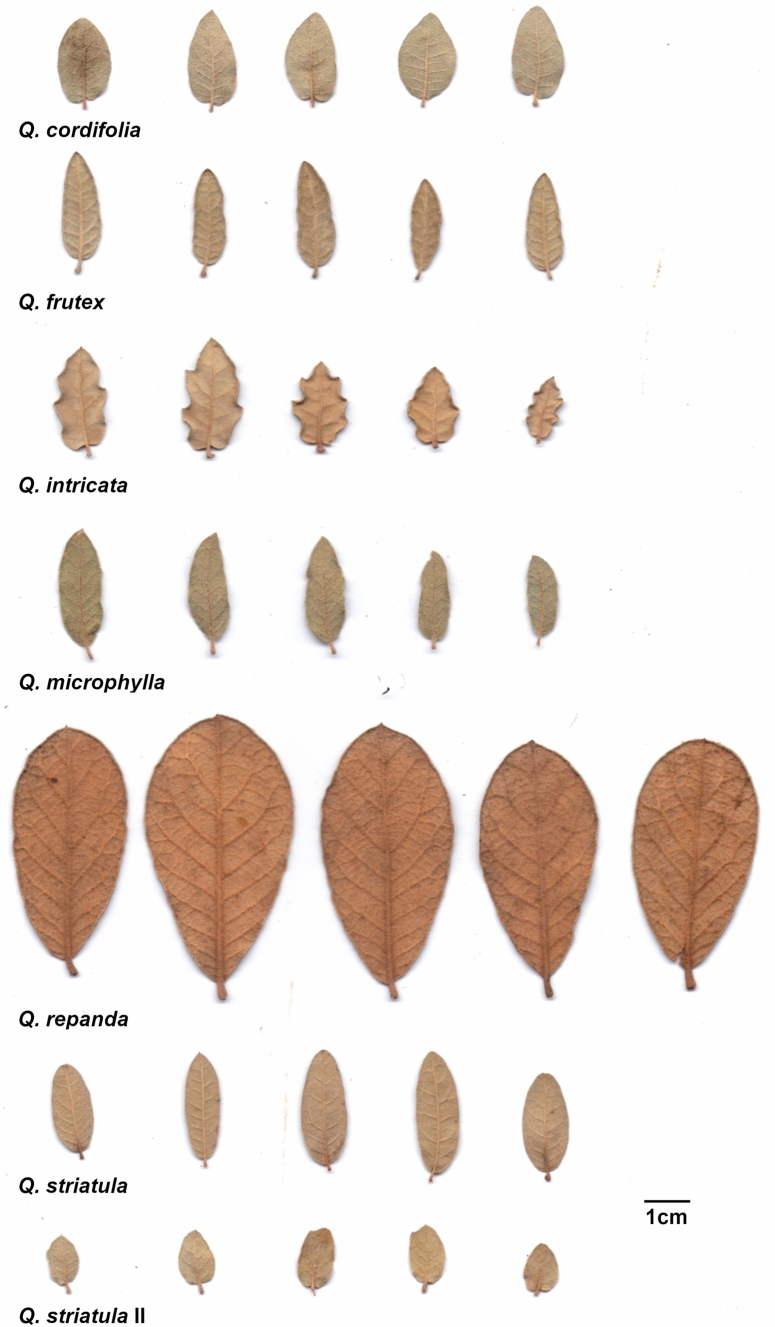
Representative leaves of the six species of the *Q. microphylla* complex on abaxial view. Leaves of the *Q. striatula* II morphotype are also included

*Quercus cordifolia* Trel. (Fig. 1) grows as clearly differentiated individuals with a stem of 0.5 to 1 m in height. The leaves of this species are papyrose, ovate, ovate-lanceolate or ovate-elliptic with a generally cordate base and rounded to acute apex, with a flat and entire or sparsely lobed margin. The underside can be from dense tomentose to glabrescent, with the tomentum formed by stellate trichomes. The species is found at elevations of 1700 to 3200 m, in pine and pine-oak forests in the north of the Sierra Madre Oriental (Sabás-Rosales et al. 2017; Valencia-A 2004).

*Quercus frutex* Trel. (Fig. 1) forms extensive patches, with stems measuring 0.6 to 1.5 m. The leaves of the species are elliptic-oblong or oblong-lanceolate with a rounded base and rounded to acute apex. The texture of the leaves is subcoriaceous, with entire and revolute margins. The underside is tomentose with stipitate-fasciculate trichomes. It occurs in the clearings of oak, pine-oak, pine or fir forests and can be the dominant species in the *Quercus* scrub of the Trans-Mexican Volcanic Belt between 2000 and 3000 m. (Valencia-A et al. 2017; Vázquez-Villagrán 2000).

*Quercus intricata* Trel. (Fig. 1) can measure from 0.4 to 1.5 m in height. This species has ovate, ovate-elliptic or elliptic-oblong leaves with rounded to slightly cordate base and acute or rounded apex. Leaf texture is coriaceous, margins are serrated and notoriously revolute. Leaf undersides are densely pubescent with stellate trichomes. This species is generally distributed in the periphery of forests at the base of mountains or in dry thickets. It is an important part of submontane scrubs, in the transition zones with rosetophylous scrub and stone pine forest, on stony soils. It is found at elevations from 1600 to 2700 m in the states of Coahuila, Nuevo León and Chihuahua (Sabás-Rosales et al. 2017; Valencia-A 2004; Villareal et al. 2008).

*Quercus microphylla* Neé (Fig. 1) has several erect stems of 0.2 to 0.7 m in height emerging from the rhizome, making it difficult to delimit individuals. The species grows in dense but not large patches. The leaves are elliptic-lanceolate or oblong-lanceolate with rounded or obtuse base, acute to slightly rounded apex, subcoriaceous texture, flat to slightly revolute margin and tomentose to slightly glabrescent underside with stellate trichomes. It is found in xerophytic shrublands and pine forests, on limestone soils or less frequently on rocky soils, at elevations between 2200 and 2500 m. It is known from the center and east of the Trans-Mexican Volcanic Belt, from the states of Guanajuato, Hidalgo, Puebla, Tlaxcala and Veracruz (Valencia-A 2004; Vázquez-Villagrán 2000).

*Quercus repanda* Bonpl. (Fig. 1) also has several erect stems of 0.2 to 1.5 m in height that emerge from the rhizome, making it difficult to delimit individuals. Leaves are obovate or elliptic-oblong, with a cuneate to rounded base and obtuse to rounded apex, coriaceous texture and revolute-repanding margin. The underside is tomentose with sessile fasciculate trichomes. It grows as small islands in the clearings of pine and pine-oak forests and as a scarce element in xerophytic shrublands, at elevations of 2,100 to 3,140 m. It is mainly distributed in the Trans-Mexican Volcanic Belt, in the states of Guanajuato, Hidalgo, Michoacán, Puebla, Querétaro, San Luis Potosí and Veracruz (Valencia-A et al. 2017).

*Quercus striatula* Trel. (Fig. 1) is also characterized by erect stems of 0.3 to 1.2 m in height that emerge from the rhizome. It grows as scattered islands and covers large surfaces at the edges of forests or in clearings. It has elliptic-lanceolate or oblong-lanceolate leaves with rounded to obtuse base and acute to rounded apex. The texture of the leaves is subcoriaceous and the margin is flat to slightly revolute with tomentose undersides with stellate trichomes. It is generally associated with xerophilous scrub or pine forests, between 2,100 and 3,000 m of elevation. It is distributed on the inner slopes of the Sierra Madre Occidental, in the states of Chihuahua and Durango (Sabás-Rosales et al. 2017).

### Collection of botanical material and grouping of populations

**Fig. 2:**
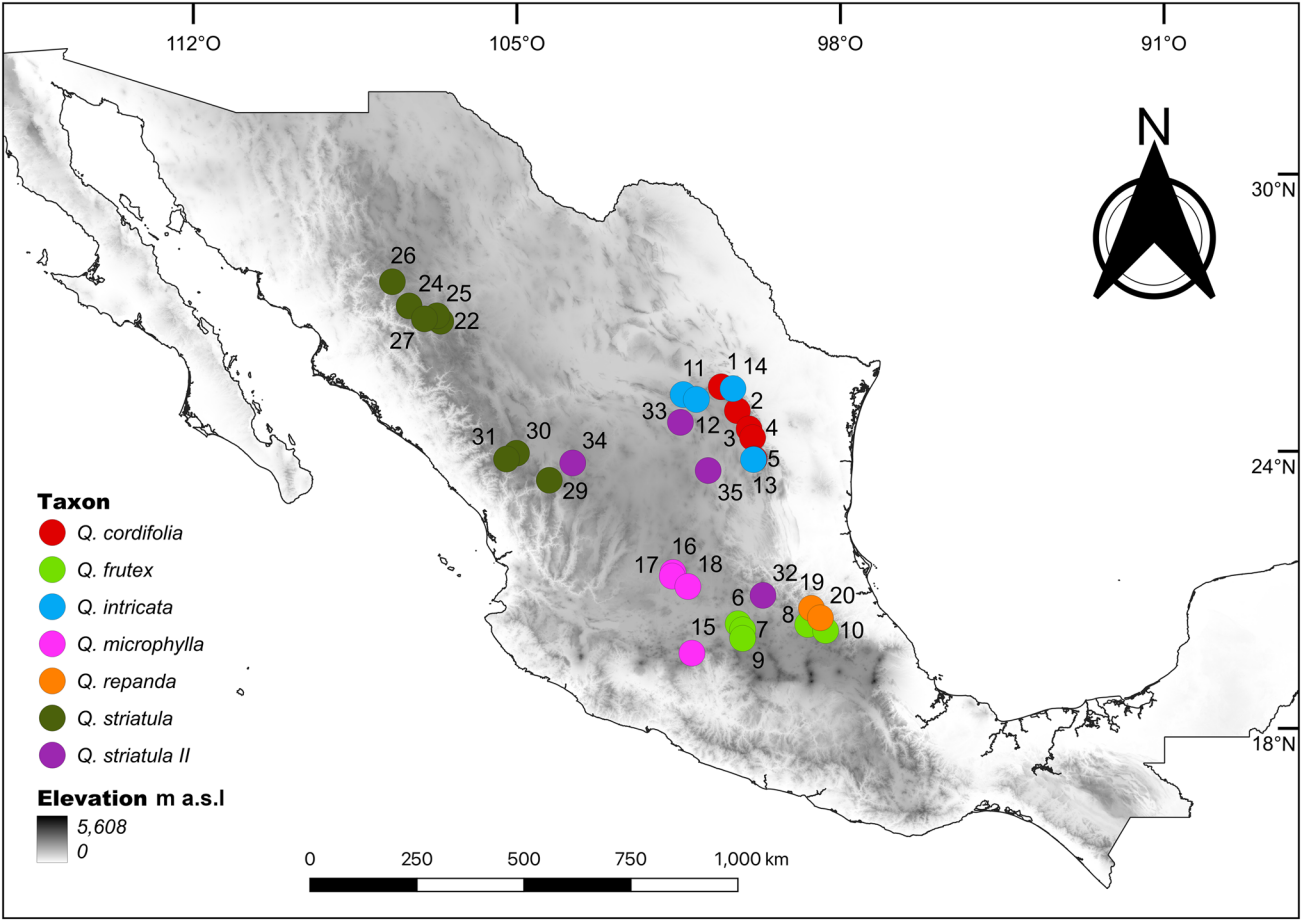
Collection sites of the populations of the *Q. microphylla* complex, accompanied by the population number according to Table 1. Gray shading indicates elevation (m a.s.l.)

To determine the distribution of the species and to select the collection localities for the different populations, we revised the specimens determined under the names *Q. cordifolia*, *Q. frutex*, *Q. intricata*, *Q. microphylla*, *Q. repanda* and *Q. striatula* deposited in the National Herbarium of Mexico (MEXU) and the herbarium of the Faculty of Sciences (FCME), both belonging to the National Autonomous University of Mexico (UNAM) (Thiers 2016). Specimens that had geographic coordinates and concurred with the original descriptions of the species and with the type specimens consulted in the electronic resource JSTOR Global Plants (plants.jstor.org) were selected. From this survey, 35 localities were chosen and sampled for the six species (Fig. 2, Table S1); also taking into account accessibility and safety aspects. During the field work, in some populations we observed variations in growth form and leaf morphology, texture and indumentum, characteristics that deviated from those reported in the original descriptions of *Q. striatula*, so these populations were designated as a variant “morphotype” (hereafter called *Q. striatula* II) (see Fig. 1). For each of the collected populations, five leaves per individual (from different parts of the shrub) were selected for all sampled individuals at the population (5–20), ensuring that selected leaves were mature and without noticeable herbivory marks. Subsequently, the leaves were pressed and dried.

### Morphological analyses

*Geometric morphometrics.* Once dried, leaves were digitized using a flatbed scanner (Hp SJ300) at a resolution of 300 dpi, always accompanied by a size scale. In order to represent the leaf shape, we placed two landmarks at homologous points of each leaf (the insertion of the petiole with the lamina, and the apex), as well as 35 equidistant points on the leaf outline (Fig. S1). Then, the tpsDig2.10 (Rohlf 2006) software was used to generate a data matrix (*x*, *y* coordinates of each of the 37 total landmarks) in “.TPS” format.

From the “.TPS” file, the matrix of coordinates was imported into the Geomorph 4.0.3 (Adams et al. 2016) package of the R Statistical Software (v4.1.2; R Core Team 2021), where the first and last points of each leaf outline were removed to avoid overlap with the apical and basal landmarks. Subsequently, a Generalized Procrustes Analysis (GPA) was implemented to remove size, position, and rotation effects. From the resulting datasets, leaf shape was averaged for each individual using the *mshape* function of the Geomorph package. In order to visualize the clustering patterns of individuals while maximizing the variance between a priori defined groups (i. e. the six species plus the *Q. striatula* II morphotype), a canonical variate analysis (CVA) was performed using the *CVA* function implemented in the Morpho 2.11 package (Schlager 2017). Additionally, subsequent CVAs were performed to explore differences within the main groups of individuals segregated by the previous analysis, this time averaging leaf shape for each population. Finally, linear distances between different pairs of landmarks (i. e. blade length, middle blade width, apical width and basal width) were calculated (Fig. S2), to analyze in a traditional way the leaf morphological differences between the taxa, using the *interlmkdist* function of the Geomorph package. Boxviolin diagrams were used to represent values of each of these linear distances, grouped by taxon. Differences among taxa were evaluated using the Kruskall-Wallis test, followed by a Dunn test as a *post-hoc* test (due to the non-normality of the data), implemented in the R software.

*Trichome imaging.* Trichomes were observed in two randomly chosen individuals from each population collected. Trichomes were scraped from the abaxial side, always from the central part of the leaf blade in the region between secondary veins and mounted on a microscope slide using fast-drying nail polish. Subsequently, these preparations were observed under an optical microscope (Zeiss Axio Imager.A2) with a 10 × objective, and images of the trichomes were digitalized and scaled using an integrated photographic camera for further analysis. From each preparation, ten trichomes were subsequently randomly selected, considering both zenithal and lateral views, to clearly observe the number of arms and their pattern of insertion. Trichomes were characterized according to the different types proposed by Jones (1986). In addition, the number of arms of each trichome was counted and a Kruskall-Wallis test, followed by a Wilcoxon test, was applied to evaluate possible differences between taxa, using the R software.

### Ecological niche models

Ecological niche models (ENM) were constructed considering seven entities: i. e. the six species and the *Q. striatula* II morphotype (Table S2). Presence records for each entity were obtained from our own field collections and specimen data from FCME and MEXU herbaria; the final dataset consisted of 15 records for *Q*. *cordifolia*, 14 for *Q*. *frutex*, 31 for *Q*. *intricata*, five for *Q*. *microphylla*, 13 for *Q*. *repanda*, 11 for *Q*. *striatula*, and 27 records for the *Q. striatula* II morphotype. To avoid overfitting the models, the *thinning* function of the spThin package (Aiello-Lammens et al. 2015) in R software was used, which randomly selects presence points separated by at least 5 km, using 100 replicates.

The 19 bioclimatic variables were obtained from the Worldclim dataset at a spatial resolution of 30 arcseconds (Fick and Hijmans 2017); https://worldclim.org/data/worldclim21.html) for the area corresponding to Mexico. These layers were trimmed into smaller areas to be used in the analyses for each taxon. The criterion for trimming was to reduce the layers according to the accessible area (area M) for each taxon, which was defined based on the ecoregions file of the World Wildlife Fund (Olson et al. 2001), selecting those ecoregions that coincided with the location of the collection sites for each taxon, as well as the areas contiguous to them, where they could hypothetically be present (Martinez-Meyer 2005). Thus, the resulting areas were used as a “*clip*” to trim the set of bioclimatic layers that were used to elaborate the ENM for each taxon. The selection and editing of the ecoregions, as well as the clipping and transformation of the bioclimatic layers to ASCII format, were performed using the QGis 3.8.3 program (QGIS Development Team 2019).

Because using a large number of bioclimatic layers can lead to overfitting of the models (Peterson and Nakazawa 2008), a pre-selection of bioclimatic variables was performed before making the final ENM. Initially, the 19 climatic variables were considered to obtain the distribution model for each taxon. From the models obtained and based on the Jackknife test of MaxEnt ver. 3.4.1 k (Phillips et al. 2006), those variables with the highest percentage of contribution to the model for each taxon were selected. Subsequently, by means of a Pearson correlation analysis in R software, from each pair of highly correlated variables (*r* > 0.80) one was eliminated, thus obtaining the sets of variables for each of the taxa (Table S2).

The ENM were performed with the maximum entropy algorithm in Maxent version 3.4.1 k (Phillips et al. 2006), since it has been demonstrated with different simulations that this algorithm generates good predictions, even with a reduced number of records (< 10; Pearson et al. 2007; Phillips et al. 2006). The parameters for the development of the models were those that came by default in the program (Phillips and Dudík 2008), except for the *Extrapolate* and *Do clamping* options that were deactivated to avoid artificial extrapolations in the extreme values of the ecological variables (Elith et al. 2011). The model output format was logistic, and 75% of the records was used to train the models and the remaining 25% to validate them; this partitioning was done randomly. Initially, the area under the curve (AUC) value of the receiver operating characteristic (ROC) was used to evaluate the robustness of the ENM. Those models with AUC values between 0.8–0.9 were considered reasonably good and ENM with values above 0.9 were categorized as very good (Peterson et al. 2011). However, considering that the usefulness of ROC analysis has been questioned (Lobo et al. 2008; Peterson and Nakazawa 2008), the final ENM were also validated by partial ROC analysis, which has been designed to address the shortcomings of ROC analysis (Peterson et al. 2008). This analysis was performed using the online tool NicheToolBox (http://shiny.conabio.gob.mx:3838/nichetoolb2/) with 1000 replicates and an omission ratio (E) = 0.05.

Finally, the degree of climate niche differentiation among taxa was measured via Schoener's *D* (Schoener 1968), using the niche similarity test implemented in ENM Tools (Warren et al. 2010, 2021). This test is recommended when species to be compared are partially or fully allopatric (Warren et al. 2010, 2021) and employs randomization to compute a null distribution and estimate whether the ENMs from different species are more or less like each other than expected by chance, based on environmental differences in the environment in which they occur (Warren et al. 2008). The test was run using the “background.test” function in R package “ENMTools” (Warren et al. 2010, 2021) with 100 replicates, sampling a total of 10,000 random points and was analyzed with a two-tailed test.

## Results

### Geometric morphometrics

**Fig. 3:**
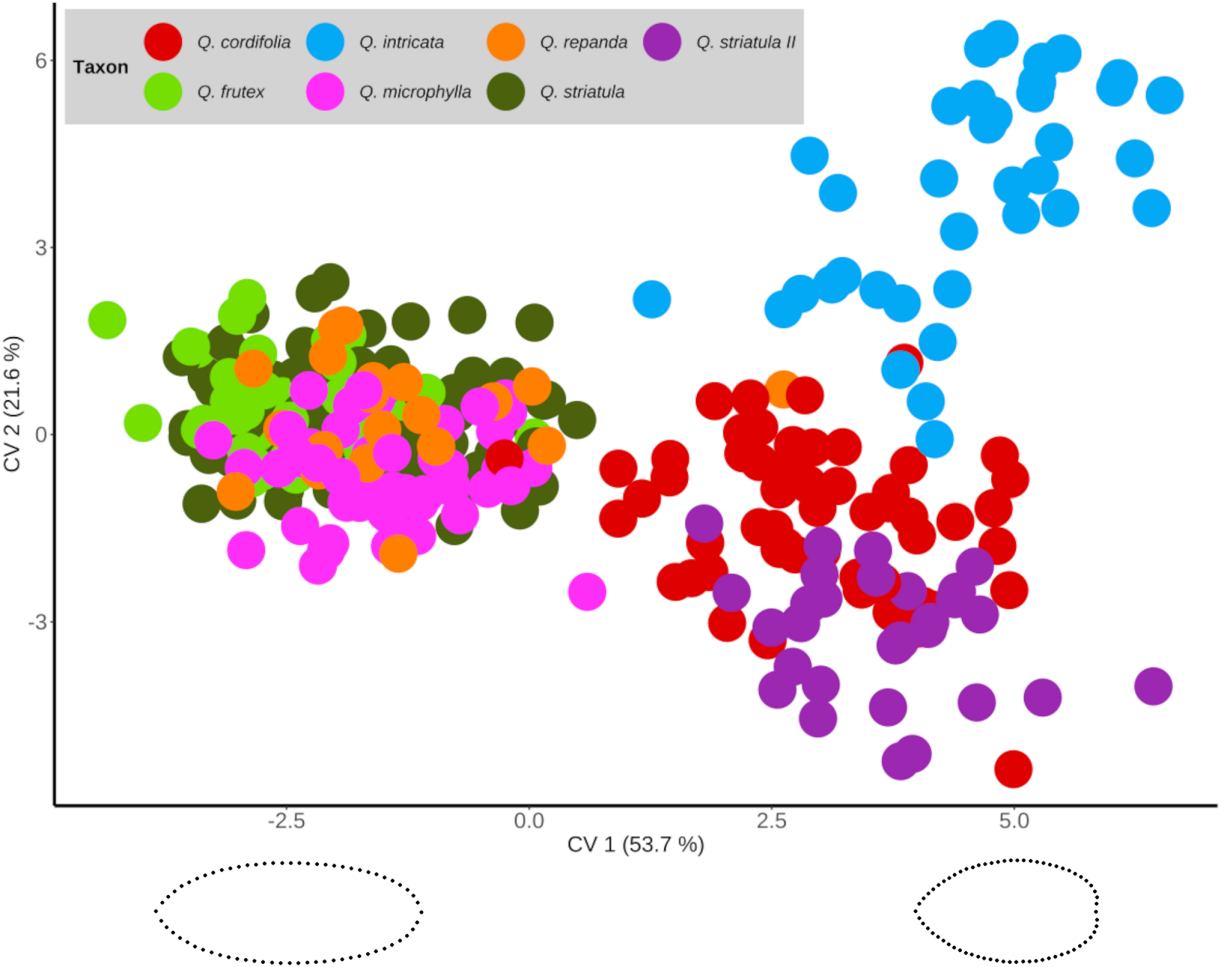
Scatterplot of the first two canonical variates (CV1 = 45.9%, CV2 = 18.7%). Each point represents the average leaf shape of an individual. Representative morphologies of the two main groups along the first canonical axis are shown at the bottom

The canonical variate analysis among all individuals indicated that 75.3% of the total variance in leaf shape was explained by the first two axes (CV1 = 53.7%, CV2 = 21.6%). The scatter plot of the first two canonical variates revealed that it is possible to distinguish two main groups separated along the first axis (Fig. 3). These groups differ significantly according to the calculated Mahalanobis distances between taxa (Table 1). The first of these two groups (Group I) is situated at negative values of the first axis and is formed by individuals of *Q. frutex*, *Q. microphylla*, *Q. repanda* and *Q. striatula*, all of which possess elliptic-oblong leaf morphologies. Meanwhile, the second group (Group II) is located towards positive values of the same axis and included individuals of *Q. cordifola*, *Q. intricata* and *Q. striatula* II, characterized by ovate leaves with a slightly cordate base (Fig. 3). Interestingly, these groups have a geographic correspondence. Populations composing Group I are distributed mainly on the Trans-Mexican Volcanic Belt (TMVB), the Sierra Madre Occidental (SMOc) and a little portion of the south of the Mexican Altiplano (MA), while populations in Group II are mainly found over the Sierra Madre Oriental (SMOr) and the Mexican Altiplano (MA) (Fig. 2).

**Table 1:** Mahalanobis distances between group means from a Canonical Variate Analysis (CVA) of individual mean leaf shape in the *Q. microphylla* complex

*Quercus* species	*Q. cordifolia*	Qf	Qi	Qm	Qr	Qs
*Q. frutex* (Qf)	**6.14**					
*Q. intricata* (Qi)	**5.75**	**8.02**				
*Q. microphylla* (Qm)	**5.26**	**3.68**	**7.73**			
*Q. repanda* (Qr)	**5.92**	**4.30**	**7.89**	**4.70**		
*Q. striatula* (Qs)	**5.46**	**2.84**	**7.38**	**3.08**	**4.22**	
*Q. striatula* (QsII)	**3.96**	**7.53**	**7.28**	**6.65**	**7.47**	**6.70**

Values in bold are significant (*P* < 0.05)

**Fig. 4:**
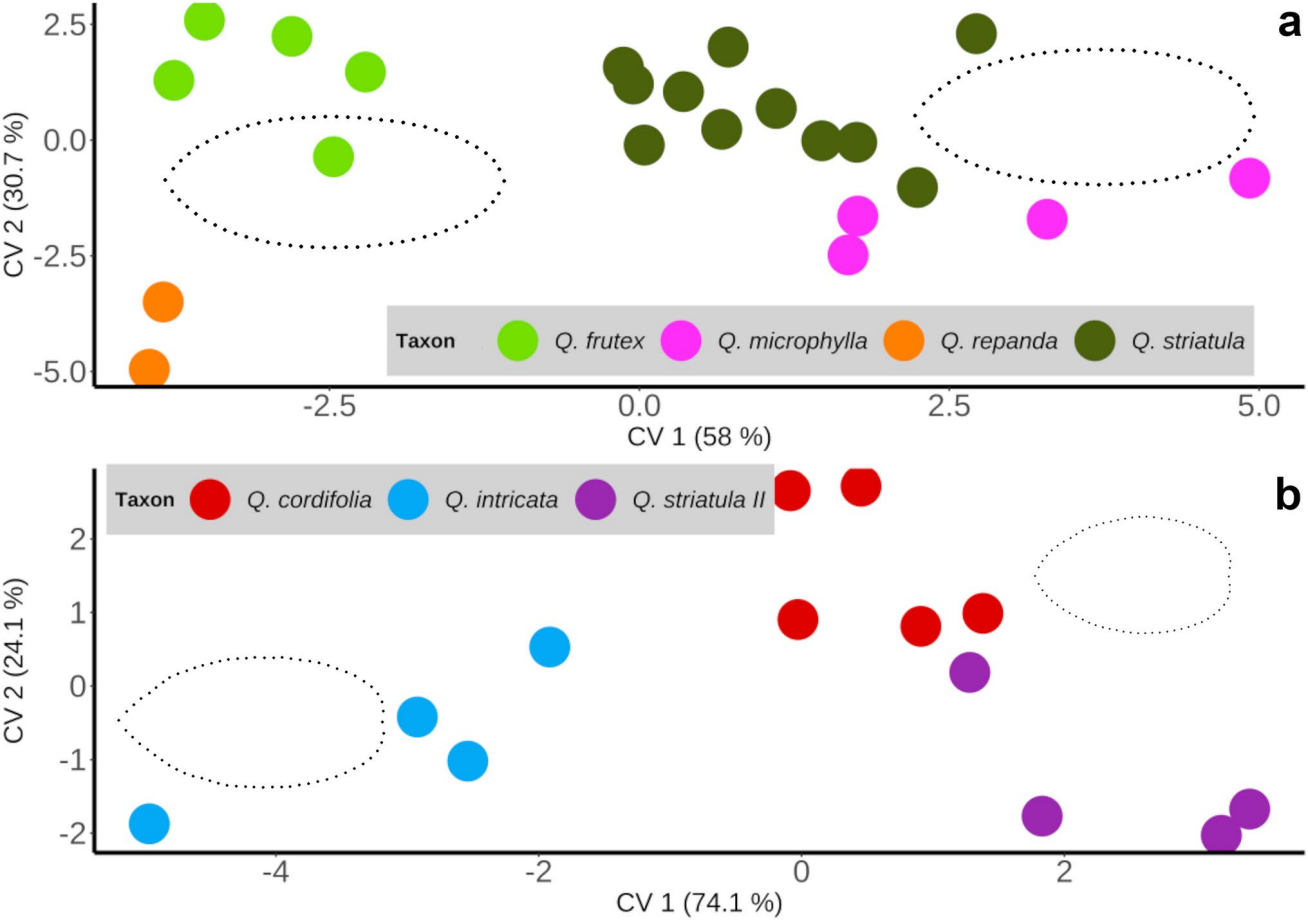
Scatterplot of population averages for the first two canonical variates corresponding to the morphological Group I (**a**) and morphological Group II (**b**). Representative leaf morphologies of the two main groups defined by the CV1 in each analysis are shown

Subsequent canonical variate analysis for the population averages of the samples belonging to Group I explained 88.7% of the variance on the first two axes (CV1 = 58%, CV2 = 30.7%). The scatter plot suggested two main morphological groupings within this group, one formed by *Q. frutex* and *Q. repanda* (with negative values along the CV1), with elliptic-oblong leaves with a slightly attenuated base. On the other hand, *Q. microphylla* and *Q. striatula* (with positive values along the CV1) formed another grouping with predominantly elliptic blades with a rounded base (Fig. 4a). However, *Q. frutex*-*Q. repanda* and *Q. striatula*-*Q. microphylla* did not differ significantly according to Mahalanobis distances (Table 2).

**Table 2:** Mahalanobis distances between group means from CVA of population mean leaf shape within the morphological Group I and morphological Group II in the *Q. microphylla* complex

Group 1			
	*Q. frutex*	*Q. microphylla*	*Q. repanda*
*Q. microphylla*	**6.65**		
*Q. repanda*	6.10	**7.67**	
*Q. striatula*	**4.42**	3.80	**6.95**

Group 2		
	*Q. cordifolia*	*Q. intricata*
*Q. intricata*	**4.29**	
*Q. striatula* II	3.50	**5.55**

Values in bold are significant (*P* < 0.05)

Likewise, in the case of Group II, the CVA analysis recovered 99% of the variation in the first two axes (CV1 = 74.1%, CV2 = 25.1%). In this case, it segregated the populations of *Q. intricata*, characterized by elliptic-ovate forms with attenuated apex and slightly cordate base, with respect to a second morphological group formed by *Q. cordifolia* and the *Q. striatula* II morphotype, both with ovate leaf blades with cordate base (Fig. 4b). In this case, the comparison between *Q. cordifolia* and the *Q. striatula* II morphotype was not significant in terms of Mahalanobis distances (Table 2).

**Fig. 5:**
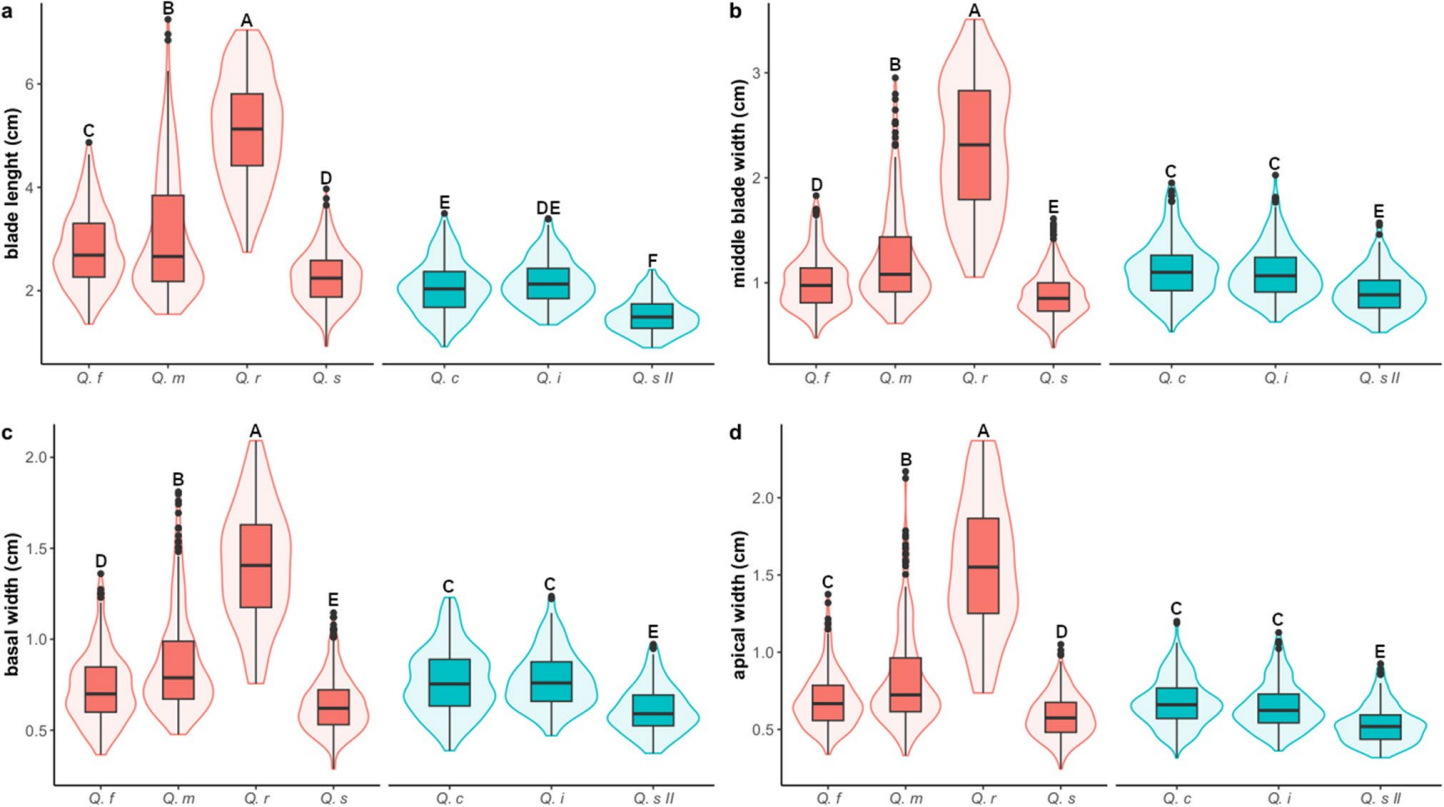
Box-violin plots of linear distances: **a** blade length, **b** middle blade width, **c** basal width, **d** apical width. Colors correspond to the two main morphological groups in Fig. 3, with red indicating taxa included in Group I and blue indicating taxa in Group II. Different letters indicate significant differences among groups according to a Kruskal–Wallis test followed by a Dunn test. Q. f = *Q. frutex*, Q. m = *Q. microphylla*, Q. r = *Q. repanda*, Q. s = *Q. striatula*, Q. c = *Q. cordifolia*, Q. i = *Q. intricata*, Q. s II = *Q. striatula* II

The distribution of the values obtained for the linear distances between pairs of landmarks reaffirms the distinctness of most taxa in pairwise comparisons for each variable (Fig. 5). Despite the similarities in leaf shape among the taxa within each of the two morphological groups, the comparisons of their linear dimensions were significant in most cases. In general, the pair of taxa with more discrimination difficulties with these variables is *Q. cordifolia*-*Q. intricata* which showed the least differences (Fig. 5).

### Trichome analysis

**Fig. 6:**
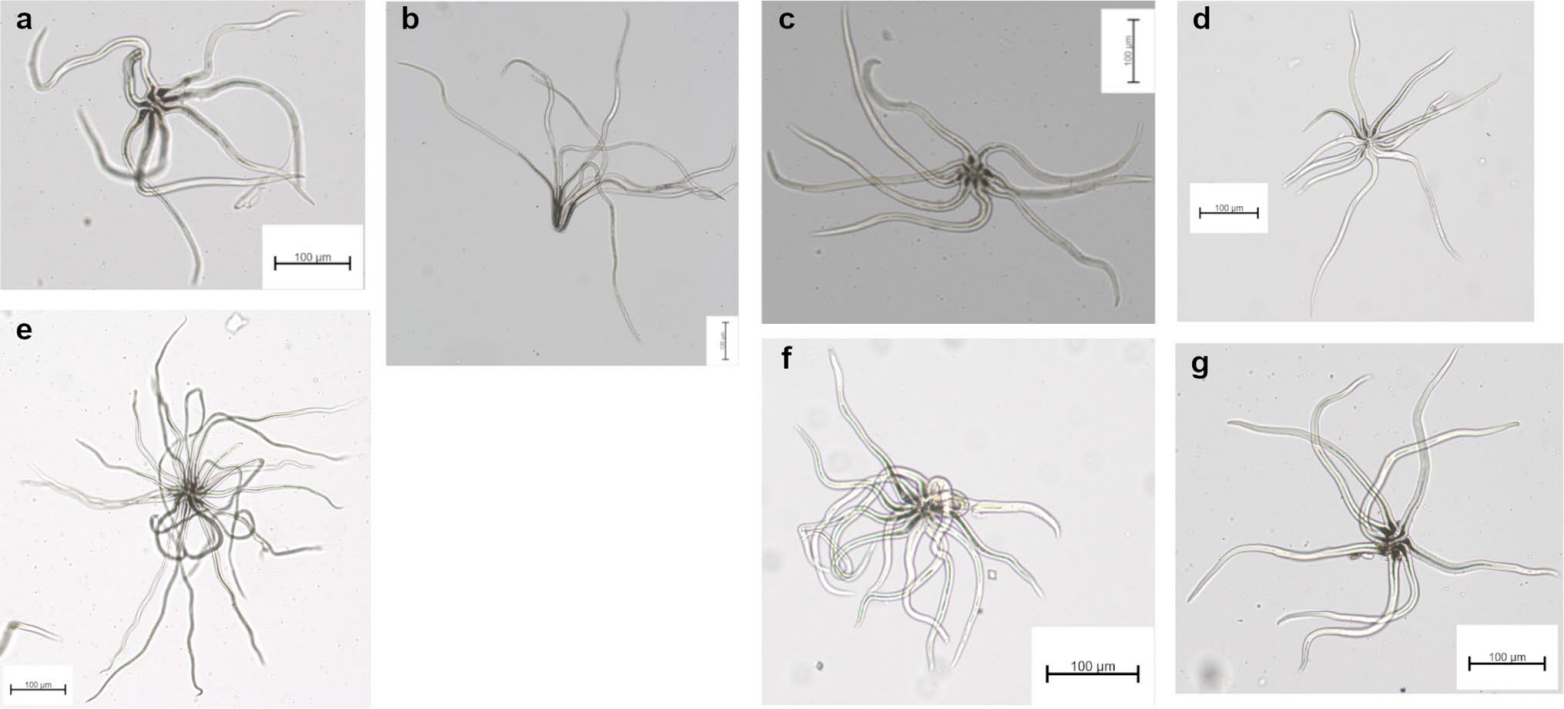
Trichomes found in the different taxa. All trichomes are of the stellate type with different number of arms, except in *Q. frutex* which shows stipitate-fasciculate trichomes. **a** *Q. cordifolia*, **b** *Q. frutex*, **c** *Q. intricata*, **d** *Q. microphylla*, **e** *Q. repanda*, **f** *Q. striatula* and **g** *Q. striatula* II

Stellate-type trichomes were found in all populations analyzed, with presence of stipitate fasciculate ones in *Q. frutex* and very scarcely in *Q. intricata* and *Q. microphylla* (Fig. 6). The number of arms of the trichomes ranged from five to 23 with a mean of eight trichomes for most taxa. The Kruskall-Wallis test (*χ*^2^ = 81.217, *P* = 0.0001) followed by a Wilcoxon post-hoc test indicated significant differences in arm number between *Q. cordifolia*-*Q. striatula*, *Q. microphylla*-*Q. striatula* and *Q. repanda* from all the other taxa (Fig. S3).

### Ecological niche models

From the Jackknife analysis and Pearson’s test, a total of two (*Q. cordifolia* and *Q. repanda*) to seven (*Q. striatula* II) non-collinear bioclimatic variables were chosen to perform the ENM of the different taxa (Table S2). In general, the ENM for the analyzed taxa were categorized as “very good” based on the AUC values, which were greater than 0.9, except for the model of *Q. repanda*, which had AUC = 0.89 for the training data and AUC = 0.86 for the validation data and was thus considered as “reasonably good”. Likewise, partial ROC tests yielded AUC ratios with values significantly (*P* < 0.0001) greater than 1 (Table S3), indicating that the models obtained are statistically better than chance.

**Fig. 7:**
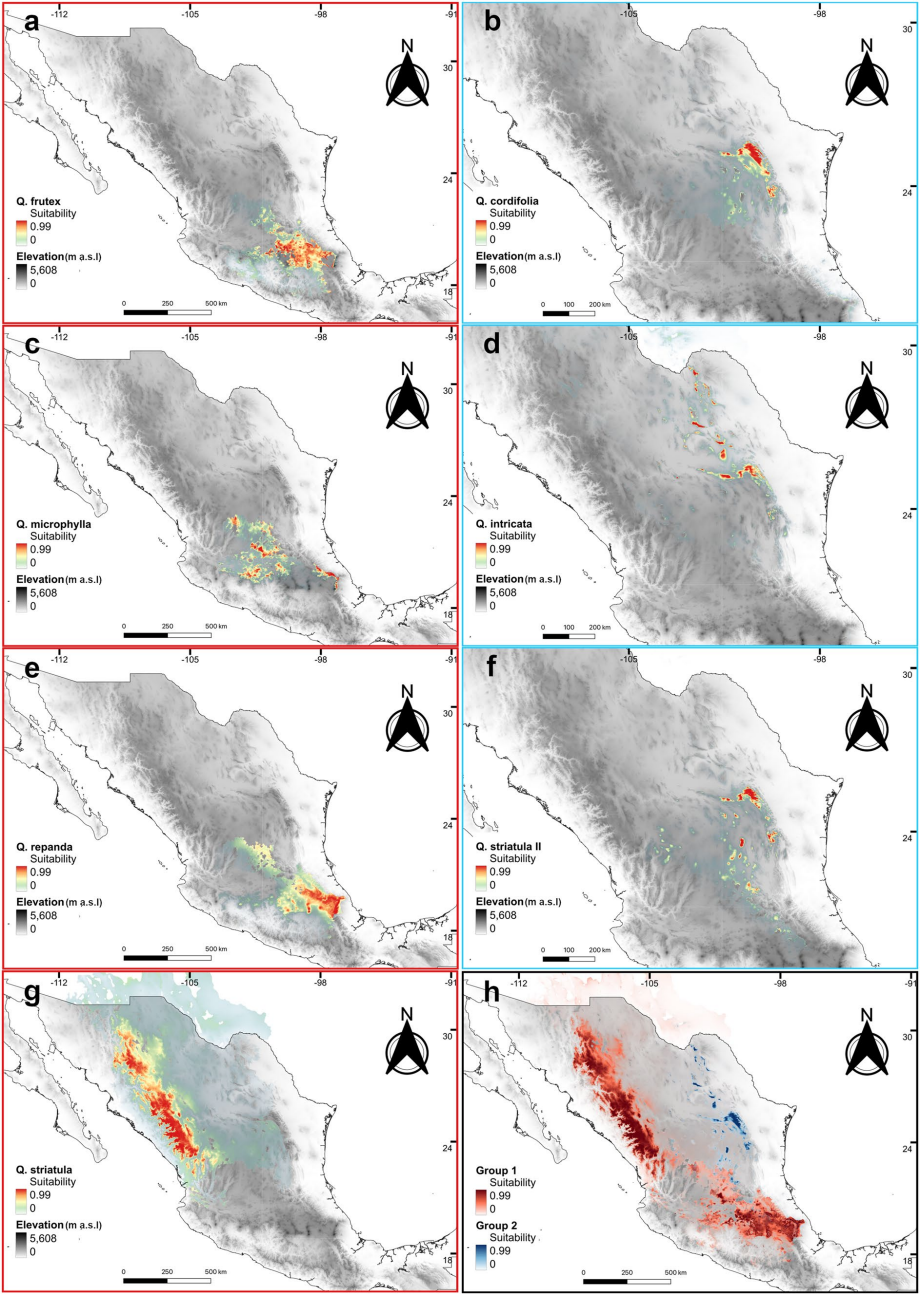
Potential distribution of seven taxa of Mexican shrub oaks. **a** *Q. frutex*, **b** *Q. cordifolia*, **c** *Q. microphylla*, **d** *Q. intricata*, **e** *Q. striatula*, **f** *Q. striatula* II, **g** *Q. repanda* and **h** distribution of two main morphological groups (red = morphological Group I; blue = morphological Group II). Gray shading indicates elevation (m a.s.l)

The potential distribution maps based on the ENM showed three main geographical patterns: (i) taxa with higher suitability in the TMVB (*Q*. *frutex, Q. microphylla* and *Q*. *repanda*); (ii) taxa with higher suitability in the SMOc (*Q*. *striatula*); and (iii) taxa with higher suitability in the SMOr and MA (*Q*. *cordifolia*, *Q*. *intricata* and *Q*. *striatula* II) (Fig. 7). These patterns also coincided with the vegetation type where the different taxa were collected: oak forest margins (TMBV), pine-oak forest clearings (SMOc), scrub and pine-oak forest (SMOr), and xerophytic scrub with pine (MA).

Compatible with what was observed with the morphological groupings, *Q. frutex*, *Q. microphylla* and *Q. repanda* (Fig. 7a, c, g) are codistributed in some parts of the TMBV, particularly towards the central part. However, the distribution of *Q. frutex* and *Q. repanda* is mostly located towards the east, while *Q. microphylla* shows a fragmented distribution mainly towards the western part of the TMBV. The fourth species of this group, *Q. striatula*, shows a potential distribution restricted to the SMOc (Fig. 7e). On the other hand, the species in the morphological Group II showed similar potential distribution areas. *Quercus striatula* II (Fig. 7f) showed a discontinuous distribution in the highlands of Nuevo León state and southern Coahuila state, similar to what was observed for *Q. cordifolia* (Fig. 7b). *Quercus intricata* (Fig. 7d), although sympatric with these two previous species in some areas of the SMOr, is also found discontinuously in central and northern Coahuila and even reaches the United States of America, according to records not used in this work. The pairwise niche comparison tests were significant in all cases, with Schoener’s *D* values ranging between 0.069 and 0.688 (Table 3). The lowest *D* value was obtained between *Q*. *striatula* and *Q*. *striatula* II, revealing that the niche occupied by the two taxa are highly divergent from each other (*D* = 0.069; *P* = 0.009). In turn, the highest niche similarity was observed between *Q*. *frutex* and *Q*. *repanda* (*D* = 0.688: *P* = 0.029).

**Table 3:** Results of pairwise niche comparisons tests based on Schoener’s *D* for the seven shrub oak taxa in the *Quercus microphylla* complex based on 100 replicates

*Quercus* species pairwise comparison	Range overlap	Schoener’s D	*P*-value Background test
*Q. cordifolia*-*Q. frutex*	Allopatric	0.191	0.009
*Q. cordifolia*-*Q*. *intricata*	Parapatric	0.472	0.009
*Q. cordifolia*-*Q*. *microphylla*	Allopatric	0.680	0.039
*Q. cordifolia*-*Q*. *striatula*	Allopatric	0.168	0.009
*Q. cordifolia- Q*. *striatula* II	Parapatric	0.556	0.009
*Q. cordifolia- Q*. *repanda*	Allopatric	0.471	0.009
*Q. frutex*-*Q*. *intricata*	Allopatric	0.077	0.009
*Q. frutex*-*Q*. *microphylla*	Parapatric	0.355	0.009
*Q. frutex*-*Q*. *striatula*	Allopatric	0.109	0.009
*Q. frutex-Q*. *striatula* II	Allopatric	0.129	0.009
*Q. frutex- Q*. *repanda*	Parapatric	0.688	0.029
*Q*. *intricata-Q*. *microphylla*	Allopatric	0.388	0.029
*Q*. *intricata- Q*. *striatula*	Allopatric	0.098	0.009
*Q*. *intricata-Q*. *striatula* II	Parapatric	0.461	0.009
*Q*. *intricata-Q*. *repanda*	Allopatric	0.276	0.009
*Q*. *microphylla-Q*. *striatula*	Allopatric	0.296	0.009
*Q*. *microphylla-Q*. *striatula* II	Allopatric	0.284	0.009
*Q*. *microphylla-Q*. *repanda*	Parapatric	0.568	0.019
*Q*. *striatula*-*Q*. *striatula* II	Allopatric	0.069	0.009
*Q*. *striatula*-*Q*. *repanda*	Allopatric	0.275	0.009
*Q*. *striatula* II*-Q*. *repanda*	Allopatric	0.209	0.009

## Discussion

Discrimination and circumscription of species at the morphological and molecular level, as well as their biogeographical and ecological characterization, is one of the current challenges prevailing for several species groups within the genus *Quercus* (Denk et al. 2017). However, this challenge is increased in groups of recently diverged species (Pinheiro et al. 2018), being particularly complicated when diversification or lineage formation occurs with few morphological changes (cryptic or sister species), or when reduced differentiation results from non-adaptive radiation, morphological stasis, or phenotypic convergence (Bickford et al. 2007; Morales-Saldaña et al. 2022; Nosil and Feder 2012; Valencia-A et al. 2016; Valencia-A 2020).

### Leaf analysis from a population perspective, the first part of the puzzle

Recently, the need to adopt methodologies for taxonomic purposes based on data from several individuals and different populations has been highlighted, mainly due to the variation that individuals or populations may present (Di Pietro et al. 2020). Such variation arises as a result of phenotypic plasticity or local adaptation to the different environments in which populations develop, sometimes leading to the formation of ecomorphotypes, potentially causing a taxonomic overestimation of the number of species (Di Pietro et al. 2020; Proietti et al. 2021). At the other extreme, the number of species can be underestimated because of morphological similarities, as reported by Morales-Saldaña et al. (2022) for the *Q. laeta* complex, with a large number of morphotypes and synonymies within it, which may correspond to different species.

For this reason, among the most powerful tools of current morphological analysis, which has been used in different taxonomical complexes, geometric morphometrics has the advantage of providing statistical robustness, while the analysis of the shape as a whole will also implicitly evaluate different traits quantified by traditional methods (e.g., leaf length, width, distance between lobes and/or veins). In this way, a detailed analysis of the species hypotheses being tested is possible, including the identification of shape aspects for which the evaluated groups differ the most, therefore contributing to the elaboration of an overall diagnosis for each group of samples from a population perspective (Pérez-Pedraza et al. 2021; Valencia-Cuevas et al. 2015; Viscosi et al. 2009a, 2010; Yang et al. 2022).

The *Q. microphylla* complex has been reviewed in few taxonomic works. Trelease (1924) proposed the *Microphyllae* series, at that time only consisting of the currently recognized *Q. microphylla*, *Q. frutex* and *Q. repanda*. However, the high intraspecific morphological variation of these species generates a morphological gradation that results in an overlap of the forms reported in the original descriptions that, together with the presence of non-glandular trichomes in the indumentum of all of them, has caused diverse interpretations on the validity of the taxa (Sabás-Rosales et al. 2017).

Currently, the *Q. microphylla* complex as proposed by Valencia-A (2020) is composed of *Q. cordifolia, Quercus deliquescens, Q. frutex, Q. intricata, Q. microphylla, Quercus mohoriana, Q. repanda* and *Q. striatula*. Our geometric morphometrics results subdivided the complex into two groups (Fig. 3). On the one hand, in Group I, we found *Q. frutex*, *Q. repanda*, *Q. microphylla* and *Q. striatula*, for which the possible synonymy and identity problems between the latter and *Q. cordifolia*, previously discussed by Muller (1944); Martínez (1977) and Villareal et al. (2008), are morphologically ruled out. On the other hand, morphological similarity between *Q. frutex* and *Q. repanda* was observed (Fig. 4a), as reported by Romero et al. (2002) and Valencia-A et al. (2017). However, they differ in all linear dimensions and also in shape, from elliptic forms with attenuated base (*Q. frutex*) to obovate forms (*Q. repanda*) (Figs. 4a, 5). Finally, in this group there is similarity between *Q. microphylla* and *Q. striatula*, both with elliptical forms and rounded base, which may explain what was reported by McVaugh (1974) and González (1986).

On the other hand, it should be noted that the morphotype *Q. striatula* II, which has been determined as *Q. striatula* in herbaria, clearly differs from the type and specimens considered as *Q. striatula* in this work, partially explaining the identity problems between these taxa. In this regard, within Group II, the CVA based on population morphological averages clearly separated *Q. intricata* from *Q. striatula* II. At the same time, similarity was observed between the latter and *Q. cordifolia* (Fig. 4b), which have had identity problems according to Muller (1944) and Villareal et al. (2008) by presenting ovate leaves with a cordate base. Despite this, they differ in the four linear dimensions, with all measures being smaller in *Q. striatula* II (Fig. 5). So, apparently the taxonomic complexity of *Q. cordifolia* has actually occurred with *Q. striatula* II and not with *Q. striatula*.

Finally, the indumentum in all species presented trichomes of the stellate type, with a mean around eight arms for most of them (*Q. cordifolia, Q. frutex, Q. intricata, Q. microphylla, Q. striatula* and *Q. striatula* II) clearly separating only *Q. repanda* (mean of 19 arms) from the rest. In addition, the stipitate stellate trichome type is generally present in *Q. frutex* (Fig. 6). Therefore, these two taxa can be clearly discriminated from the rest of the complex under these two traits. Likewise, the number of arms achieved significant differences between *Q. microphylla* and *Q. striatula*, species with great similarity in morphometric measurements (Fig. 5). In this sense, it is still necessary to quantitatively evaluate the density of trichomes in the leaf lamina, as well as the epidermis type to find out if there are differences among the different taxa (Fortini et al. 2015; López-Caamal et al. 2017; Scareli-Santos et al. 2013; Tschan and Denk 2012).

### Ecological and geographical patterns within the* Q. microphylla* complex

The geographic distribution of the taxa in the *Q. microphylla* complex is one aspect of notorious disagreement among different authors (González-Elizondo et al. 1993; Romero Rangel et al. 2002; Sabás-Rosales et al. 2017; Valencia-A 2004; Valencia-A et al. 2017; Vázquez-Villagrán 2000). From a total of 21 pairwise comparisons of potential distribution models, in six comparisons (28.6%) we found some degree of overlap in the geographic ranges of the taxa, while in 15 comparisons (71.4%) they showed non overlapping distributions (Table 3). This suggests that, in most cases, allopatry is the predominant distribution pattern among species of the *Q*. *microphylla* complex. In addition, when we contrasted the potential distributions between the two main groups detected through geometric morphometry, it was also possible to observe that these two groups are largely allopatric. Species of Group I are distributed towards the center and west of the country, through the TMVB, SMOc and the southern portion of the MA, while taxa in Group II are found mainly towards the center and northeast of Mexico, in the south and north of the MA and the SMOr (Fig. 7). Therefore, this zoning, together with morphometric analyses, allows an approximation of the identity of species from their distribution, making it possible to rule out the presence of some taxa in certain regions.

Complementarily, the background test allowed us to quantify the degree of niche similarity among taxa and to incorporate ecological evidence to clarify taxonomic controversies within the *Q*. *microphylla* complex. Firstly, the climatic niche comparisons allowed us to support the hypothesis that *Q*. *striatula* and *Q*. *striatula* II actually represent two distinct biological entities, with a remarkable morphometric differentiation, a clear allopatric distribution pattern and a strong niche divergence. Thus, they could be considered as two different species. In addition, in cases in which there is a limited morphometric differentiation, as it happens between *Q*. *microphylla* and *Q*. *striatula,* the analysis indicated a high degree of niche differentiation with an evident allopatric pattern, so that the low morphological differentiation could be due to phenotypic convergence, as has been reported for other Mexican oaks (Valencia-A 2020). Even though our results indicated a tendency toward niche divergence among taxa within the *Q. microphylla* complex, examples of taxa with high degree of niche similarity were also observed, which were accompanied by morphological similarity (e. g., *Q. cordifolia*-*Q. striatula* II, *Q. frutex*-*Q. repanda* and *Q. microphylla*-*Q. repanda*). However, the different types of evidence combined allows discrimination among taxa in almost all cases.

Finally, paradoxically *Q. microphylla* is the species of the complex with the widest previously reported distribution. In spite of this, during our field work it was very difficult to find populations of this species, so apparently, the high taxonomic complexity of the group has resulted in an overestimation of the distribution of this species, mainly towards the north of the country. In addition, one of the problems detected during the herbarium revision is that several specimens were indicated to have a tree growth form in the collection data. This alternation between tree and shrub habit can occur in species such as *Quercus opaca* or *Quercus grisea*. However, the species that comprise the *Q. microphylla* complex are strictly shrubby. But, apparently the shape, and particularly the size of the leaves, generates this confusion at the moment of identifying specimens, contributing to overestimate the distribution of the species.

### *Q. microphylla*, Mexico’s largest shrub oak complex

The *Q. microphylla* complex represents the shrub oak complex with more species in Mexico (eight of at least 32 species present in the country). In addition to the species studied in the present work, *Q. deliquescens* and *Q. mohriana* are part of the complex, since they share the characteristics of shrubby habit, stellate trichomes and elliptic-oblong to ovate leaves. In the case of *Q. deliquescens*, it was not possible to locate any population corresponding to the specimens deposited in the herbaria, besides being presumably restricted to the state of Chihuahua. In turn, *Q. mohriana* is mainly distributed in the United States of America and in Mexico it is only found in the north of the state of Coahuila. Therefore, both species were not considered for the present work. However, all these species together result in a set of taxa with high morphological/taxonomic complexity within the genus *Quercus*.

Moreover, our results provide valuable information that can complement the poorly detailed descriptions of most species. And, consequently, they can serve as a basis for different types of studies in the genus. In this sense, it has been suggested to carefully analyze different evidence simultaneously (e. g. morphometric, ecological niche modeling, molecular and chemical data, etc.), with a wide representation of individuals and populations; and in this way, to assign a set of morphological characters covering the known distribution of the species to also assign its *locus classicus* (name linked to a type specimen, also linked to a geographical locality), as a way of adding additional ecological information enabling further identity of the species (Di Pietro et al. 2020).

Finally, the need for molecular analysis of species boundaries was highlighted. On the one hand, the strong separation between the morphotypes of *Q. striatula* suggests the presence of two different species (they are morphologically, geographically and ecologically distant). Therefore, the morphotype *Q. striatula* II may remain under some synonymy or be undescribed. On the other hand, *Q. microphylla* and *Q. striatula* are morphologically grouped, and show intraspecific variation that deviates from the description of each species, mainly in aspects such as leaf consistency, appearance of the indumentum and the way in which the shrub develops. Also, the similarity between *Q. microphylla* and *Q. frutex*, mainly in geographically close populations, can still generate taxonomic confusion. All these variations generate uncertainty about the existence of other unrecognized taxa within the analyzed populations. Therefore, evaluating the different species hypotheses with molecular data can greatly contribute to clarify the identity of these variants.

### Supplementary Information

Below is the link to the electronic supplementary material.

Supplementary file1 (PDF 200 KB)

## Data Availability

Original data used for this study are available upon request to the corresponding author.
