# Supplementary material for: Leaf morphometric analysis and potential distribution modelling contribute to taxonomic differentiation in the Quercus microphylla complex
Source: J Plant Res. 2023 Sep 23;137(1):3–19. doi: 10.1007/s10265-023-01495-z (PMC10764464; doi:10.1007/s10265-023-01495-z)
Supplement: Supplementary file 1 — Supplementary file1 (PDF 200 KB) [file 10265_2023_1495_MOESM1_ESM.pdf]

Electronic supplementary materials

Title:

Leaf morphometric analysis and potential distribution modelling contribute to taxonomic differentiation in the *Quercus microphylla* complex.

Authors:

Oscar Angel De Luna-Bonilla, Susana Valencia-Á, Guillermo Ibarra-Manríquez, Saddan Morales-Saldaña, Efraín Tovar-Sánchez and Antonio González-Rodríguez.

Journal:

Journal of Plant Research

Corresponding author:

Antonio González-Rodríguez

IIES UNAM

Antigua Carretera a Pátzcuaro No.8701. Col. Ex Hacienda de Sán José de la Huerta C.P. 58190, Morelia, Michoacán, México

Tel: +52 (443) 322 27 00

E-mail: **agrodrig@cieco.unam.mx**

Content:

Table S1. Collection sites and geographic information for the 35 populations of the taxa of the *Quercus microphylla* complex in Mexico.

Table S2. Bioclimatic variables used for ecological niche modeling of each taxon of the *Quercus microphylla* complex.

Table S3. Statistical results of the ecological niche models for the analyzed taxa of the *Quercus microphylla* complex.

Fig S1. Position of the placed landmarks. 1) insertion of petiole with leaf lamina, 2) apex of lamina and two curves drawn with 35 equidistant points on the leaf outline.

Fig S2. Layout of landmarks and calculated distances between pairs of landmarks for: blade length (1,2), middle blade width (19-52), basal width (8-41) and apical width (30,63).

Fig S3. Box-violin plots of number of arms for taxon. Colors correspond to the two main morphological groups in Figure 3, with red indicating taxa included in Group I and blue indicating taxa in Group II. Different letters indicate significant differences among groups according to a Kruskal-Wallis test followed by a Wilcoxon test.

Table S1. Collection sites and geographic information for the 35 populations of the taxa of the *Quercus microphylla* complex in Mexico.

Taxon/Population number	Locality(State)	Latitude	Longitude
Quercus cordifolia			
1	Lirios (Coahuila)	25.397	-100.579
2	Galeana(Nuevo León)	24.878	-100.231
3	Pablitos (Nuevo León)	24.483	-99.977
4	Ascensión (Nuevo León)	24.294	-99.901
5	Picacho (Nuevo León)	23.833	-99.865
Q. frutex			
6	Huimilpan (Querétaro)	20.264	-100.214
7	Mezquititlán(Querétaro)	20.135	-100.115
8	Santiaguito (Hidalgo)	20.25	-98.708
9	Temascalcingo (México)	19.948	-100.116
10	Tulancingo (Hidalgo)	20.119	-98.321
Q. intricata			
11	Nogal (Coahuila)	25.229	-101.402
12	Carneros (Coahuila)	25.121	-101.116
13	Onofre (Nuevo León)	23.822	-99.8807
14	Plegada (Nuevo León)	25.356	-100.325
Q. microphylla			
15	Piedra (Michoacán)	19.629	-101.213
16	Lobos (Guanajuato)	21.375	-101.622
17	Villa Seca (Guanajuato)	21.297	-101.64
18	Guanajuato (Guanajuato)	21.072	-101.3
Q. repanda			
19	Huayacocotla (Hidalgo)	20.6	-98.626
20	Puerto (Veracruz)	20.394	-98.433
Q. striatula			
21	Aboreachi A (Chihuahua)	27.147	-107.331
22	Aguaje (Chihuahua)	26.814	-106.646
23	Guachochic A (Chihuahua)	26.866	-107.001
24	Aboreachi B (Chihuahua)	27.147	-107.331
25	Agostadero (Chihuahua)	26.933	-106.729
26	Creel (Chihuahua)	27.671	-107.693

27	Guachochic B (Chihuahua)	26.866	-107.001
28	Monjes (Chihuahua)	27.654	-107.561
29	Michilía (Durango)	23.372	-104.299
30	Otinapa (Durango)	23.958	-105.007
31	Salto (Durango)	23.831	-105.224
Q. striatula II			
32	Juárez (Querétaro)	20.879	-99.675
33	Mazapil (Zacatecas)	24.625	-101.461
34	Órganos (Zacatecas)	23.747	-103.792
35	Wadley (San Luis Potosí)	23.583	-100.862

Table S2. Bioclimatic variables used for ecological niche modeling of each taxon of the *Quercus microphylla* complex.

Species	Variable
Quercus cordifolia	Bio5, Bio10
Q. frutex	Bio4, Bio9, Bio18, Bio19
Q. intricata	Bio2, Bio3, Bio5, Bio11
Q. microphylla	Bio2, Bio3, Bio8, Bio18,
Q. repanda	Bio8, Bio15
Q. striatula	Bio2, Bio6, Bio10, Bio12, Bio19
Q. striatula II	Bio3, Bio4, Bio5, Bio14, Bio15

Table S3. Statistical results of the ecological niche models for the analyzed taxa of the *Quercus microphylla* complex.

Species	AUC Training	Test	Partial ROC AUC ratios
Quercus cordifolia	0.981	0.994	1.9707
Q. frutex	0.922	0.885	1.8544
Q. intricata	0.981	0.987	1.9628
Q. microphylla	0.932	0.972	1.9114
Q. repanda	0.893	0.860	1.7895
Q. striatula	0.943	0.950	1.9071
Q. striatula II	0.990	0.987	1.9787

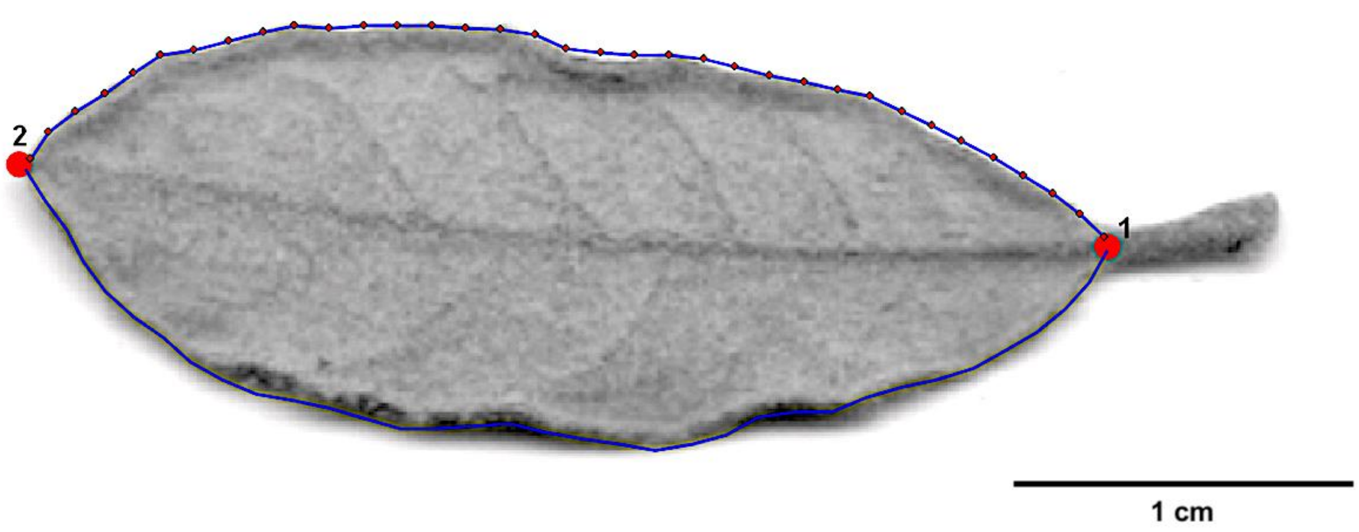

Fig S1. Position of the placed landmarks. 1) insertion of petiole with leaf lamina, 2) apex of lamina and two curves drawn with 35 equidistant points on the leaf outline.

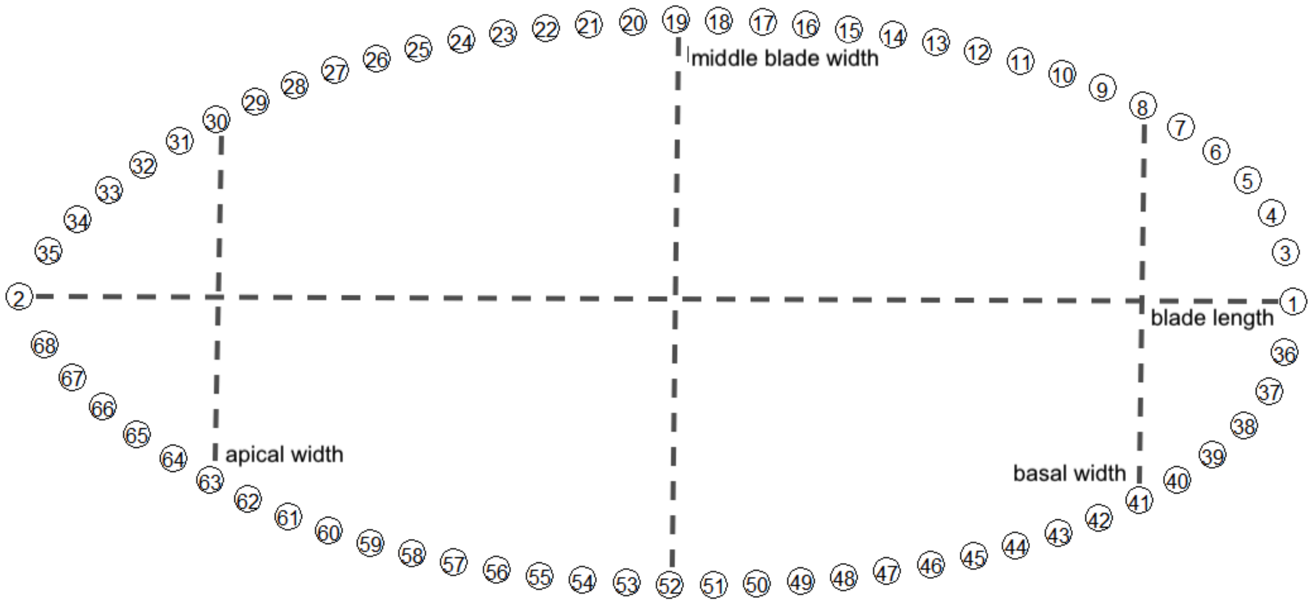

Fig S2. Layout of landmarks and calculated distances between pairs of landmarks for: blade length (1,2), middle blade width (19-52), basal width (8-41) and apical width (30,63).

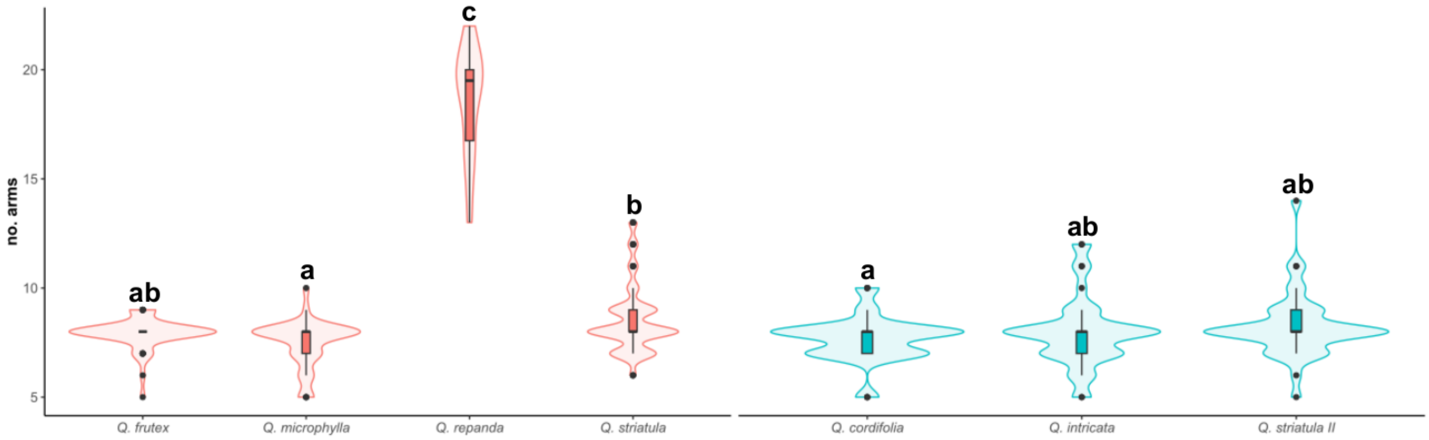

Fig S3. Box-violin plots of number of arms for taxon. Colors correspond to the two main morphological groups in Figure 3, with red indicating taxa included in Group I and blue indicating taxa in Group II. Different letters indicate significant differences among groups according to a Kruskal-Wallis test followed by a Wilcoxon test.
